# Overlapping Mechanisms of Stress-Induced Relapse to Opioid Use Disorder and Chronic Pain: Clinical Implications

**DOI:** 10.3389/fpsyt.2016.00080

**Published:** 2016-05-02

**Authors:** Udi E. Ghitza

**Affiliations:** ^1^U.S. Department of Health and Human Services (HHS), Center for the Clinical Trials Network (CCTN), National Institute on Drug Abuse (NIDA), National Institutes of Health (NIH), Bethesda, MD, USA

**Keywords:** chronic pain, addiction, opiate addiction, addiction treatment, opiate dependence, opioid dependence, substance use disorders, substance use disorder

## Abstract

Over the past two decades, a steeply growing number of persons with chronic non-cancer pain have been using opioid analgesics chronically to treat it, accompanied by a markedly increased prevalence of individuals with opioid-related misuse, opioid use disorders, emergency department visits, hospitalizations, admissions to drug treatment programs, and drug overdose deaths. This opioid misuse and overdose epidemic calls for well-designed randomized-controlled clinical trials into more skillful and appropriate pain management and for developing effective analgesics that have lower abuse liability and are protective against stress induced by chronic non-cancer pain. However, incomplete knowledge regarding effective approaches to treat various types of pain has been worsened by an under-appreciation of overlapping neurobiological mechanisms of stress, stress-induced relapse to opioid use, and chronic non-cancer pain in patients presenting for care for these conditions. This insufficient knowledge base has unfortunately encouraged common prescription of conveniently available opioid pain-relieving drugs with abuse liability, as opposed to treating underlying problems using team-based multidisciplinary, patient-centered, collaborative-care approaches for addressing pain and co-occurring stress and risk for opioid use disorder. This paper reviews recent neurobiological findings regarding overlapping mechanisms of stress-induced relapse to opioid misuse and chronic non-cancer pain, and then discusses these in the context of key outstanding evidence gaps and clinical-treatment research directions that may be pursued to fill these gaps. Such research directions, if conducted through well-designed randomized-controlled trials, may substantively inform clinical practice in general medical settings on how to effectively care for patients presenting with pain-related distress and these common co-occurring conditions.

## Introduction

Recent evidence synthesis highlights substantial brain-mechanism overlaps between opioid use disorder and psychological-distress components of chronic non-cancer pain (1, 2). Medications that block reinstatement of opioid-seeking behavior in animal models may be useful for opioid use disorder treatment, and as will be discussed later, basic research suggests that some may also have benefits for treating pain. This mini-review will present a synthesis of recent science regarding overlapping mechanisms of stress-induced relapse to opioid use that may also mediate the emotional stress-inducing components of chronic non-cancer pain. It will then discuss clinical-treatment research implications relevant to care management of patients with co-occurring opioid misuse and chronic non-cancer pain, evidence gaps, and treatment research directions that may be advanced to fill these gaps.

## Recent Translational Research Regarding Stress-Modulating Mechanism Governing Opioid-Seeking Behaviors

Chronic opioid use and chronic non-cancer pain may render the brain extended amygdala system hypersensitive to the negative emotional lows, such as dysphoria, anxiety, irritability, and hyperalgesia occurring following extended opioid use and during withdrawal (1), a psychological-distress state that heightens vulnerability to opioid-use relapse. In a reinstatement model of drug relapse, alpha-2 adrenergic receptor agonists (for example, clonidine or lofexidine) that inhibit noradrenaline/norepinephrine activity in the extended amygdala block stress-induced relapse to opioid and cocaine seeking (3, 4). Clonidine, an alpha-2 receptor agonist, has been used off-label for years in an opioid-using population to ameliorate symptoms of opioid withdrawal. Kowalczyk et al. tested clonidine for relapse prevention in opioid-dependence in the context of opioid agonist maintenance with buprenorphine (5). Perhaps alpha-2 receptor agonists combined with opioid agonist maintenance (such as buprenorphine) may be more effective than buprenorphine alone in extending abstinence from opioid use, related to a protective effect against stress-triggered opioid use. In a randomized double-blind placebo-controlled trial enrolling 118 treatment seekers with opioid use disorder, clonidine was tested for relapse prevention. Opioid treatment seekers who were maintained on buprenorphine maintenance and having attained initial abstinence – submitting opioid-free urines during weeks 5 and 6 of treatment – were randomized to either continue on buprenorphine (and placebo) (*n* = 57) or receive clonidine (0.1–0.3 mg per day) added to buprenorphine (*n* = 61). In addition, to assess real-life stress and craving, these participants received handheld computers. These prompted reporting on past-hour stress or opioid craving four times a day at randomly prompted times during participant’s waking hours. Participants receiving buprenorphine plus clonidine exhibited a significantly longer time to lapse, compared with participants receiving buprenorphine plus placebo. The definition of lapse was any opioid-positive or missed urine test. Participants receiving buprenorphine plus clonidine also exhibited a longer duration of continuous opioid abstinence compared with participants receiving buprenorphine plus placebo (34.8 versus 25.5 days, Cohen’s *d* = 0.38). Clonidine was well tolerated as an adjunct to buprenorphine. The only specific symptom more likely to be reported in the clonidine group was dry mouth (5).

Thus, a clonidine adjunct, in conjunction with buprenorphine maintenance, seems to be an effective intervention that increases duration of abstinence in this population. Interestingly, real-time ambulatory data collected through ecological momentary assessment suggested that clonidine worked in humans by partially decoupling daily-life stress from opioid craving. This was not the case in the buprenorphine plus placebo condition. Therefore, these findings are translational, relative to preclinical data (3): a medication (clonidine) identified in a preclinical model may effectively work-off label as a maintenance medication and adjunct to buprenorphine to enhance prevention of lapses to opioid use and prolong abstinence. In this study, the doses of clonidine used appeared to be safe and well tolerated in this population. For clinicians and patients, these findings are noteworthy in that they show how ecological momentary assessment results may be used to identify circumstances under which clonidine maintenance is most likely to be beneficial as an adjunct to buprenorphine maintenance: protecting against relapse-inducing properties of daily-life stress.

## Overlapping Brain Circuitry Mediating Psychological-Stress Component of Pain and Stress-Induced Opioid Use

Neurobiological processes underlying stress-induced worsening of opioid use disorder following heavy opioid use and during drug withdrawal share phenomenological similarities with aversive emotional aspects of chronic non-cancer pain (1, 2). Overlapping anti-reward anxiety-inducing neuroadaptations in the extended amygdala’s neural network [e.g., amplifying pathophysiological noradrenaline signaling, and interconnected corticotrophin-releasing factor/hormone (CRF/CRH) and dynorphin-mediated signaling] after repeated pain or opioid use and during opioid withdrawal may drive common maladaptive negative emotional responses to stress. The extended amygdala consists of interconnected structures, including the central nucleus of the amygdala, bed nucleus of the stria terminalis, and nucleus accumbens shell. Indeed, these structures contain some of the highest levels of stress neurotransmitters and hormones, such as norepinephrine/noradrenaline and CRF/CRH. As with hyperactive CRF/CRH and norepinephrine tone, excessive dynorphins and kappa-opioid receptor activation in this neural network also heighten emotional-distress responses during withdrawal from opioid use and during chronic non-cancer pain. Affective hypersensitivity to stress by an overactive extended amygdala circuitry that creates aversive emotional states may motivate persons suffering from chronic non-cancer pain or opioid withdrawal to actively seek and take opioids to alleviate their negative emotions and psychological perceptions of pain. The extended amygdala sends efferent connections to diencephalic and mesencephalic parts of the brain critically involved in emotional and motivational expression, including the lateral hypothalamus and the ventral tegmental area. Importantly, the anti-reward allostatic neuroadaptations described above may impact pain’s cross-sensitization with stress and stress-induced pathophysiology of reward circuitry maintaining addictive behaviors, following chronic opioid use. Hyperactivity of the brain stress circuits in the extended amygdala during such cross-sensitization has also been linked to accompanying decreased mesolimbic dopamine reward system activity – a loss of incentive-motivational function in the nucleus accumbens and interconnected prefrontal cortical circuitry that may also drive the negative emotional state of withdrawal that increases likelihood of relapse to opioid use and perception of pain (1, 6–10).

The similar nature of anti-reward pathophysiology in the extended amygdala increasing susceptibility to stress-related relapse to opioid use and recurrence of chronic non-cancer pain has important clinical therapeutic implications. It calls for systematic lines of research into how to effectively develop an integrative patient-centered care approach for addressing the United States public health epidemic of rising morbidity and mortality related to misuse of opioids (i.e., using multidisciplinary, collaborative care to safely and effectively manage psychological stress and pain together with common comorbid conditions, such as opioid use disorders).

## Rationale and Innovative Research Questions Regarding Patient-Centered Care Approach Addressing Stress-Reduction Together with Treating Chronic Non-Cancer Pain

Over the past two decades, a steeply growing number of persons with chronic non-cancer pain have been using opioid analgesics chronically to treat it. A subset of the millions of individuals who have chronic or persistent pain use opioids (particularly full mu-opioid receptor agonists) to treat it (11, 12). The number of opioid analgesic prescriptions has increased fourfold in the United States over the past two decades, which is concerning in light of the accompanying markedly increased prevalence of individuals with opioid-related misuse, opioid use disorders, opioid-related emergency department visits, admission to drug treatment programs, overdose deaths, and hepatitis C virus prevalence (11–22). This United States opioid misuse and overdose epidemic calls for well-designed prospective randomized-controlled trial research into more skillful and appropriate pain management and for developing effective analgesics that have lower abuse liability and are protective against stress induced by chronic non-cancer pain. This is particularly important in light of high prevalence of chronic pain patients who also suffer from co-occurring anxiety or mood disorders. Thus, research-informed strategies are sorely needed to prevent opioid overdoses and decrease opioid misuse, particularly in high-risk populations for opioid use disorders, such as patients using opioids for chronic non-cancer pain who are susceptible to stress (11, 12).

One potential solution is to advance well-designed randomized-controlled trials on analgesic comparative effectiveness of combined partial mu-opioid receptor agonists (i.e., buprenorphine, which has less abuse liability than full mu-opioid receptor agonists) and alpha-2 adrenergic receptor agonists [which have been shown to have analgesic efficacy in their own right but less abuse liability relative to full mu-opioid receptor agonists (23–30)], compared to full mu-opioid receptor agonists for treating chronic non-cancer pain. As discussed, alpha-2 adrenergic receptor agonists may confer added benefits, above and beyond mu-opioid receptor partial agonists, of protecting against stress-induced opioid misuse. For instance, an interesting research question upon which to design a comparative effectiveness research trial would be the following: among adults at risk for an opioid use disorder who are already using mu-opioid agonists to treat chronic non-cancer pain, what are comparative benefits versus harms of continuing such opioids versus switching to partial mu-opioid receptor agonist buprenorphine, either (a) alone or (b) combined with an alpha-2 adrenergic receptor agonist (e.g., clonidine, lofexidine, or guanfacine)? What is the longer-term durability (>1 year) of these outcomes related to pain, function, life satisfaction, recovery from opioid use, and quality of life? The rationale for the latter comparator would be neurobiological research, suggesting that analgesic benefit of a combination mechanism-of-action approach to treating pain. Relatively, low doses of mu-opioid receptor agonists combined with alpha-2 adrenergic receptor agonists may have synergistic pain-relieving benefits, with a better safety profile than higher doses of mu-opioid receptor agonists (31–38). For such research questions to be informative to clinicians, it would also be helpful to test the comparative effectiveness of different regimens for initiating and titrating these agents for providing maximal functional, stress-reducing, and analgesic benefits and for minimizing harm.

Cannabidiol (CBD) is another promising therapeutic target that merits systematic, rigorous clinical research on its effectiveness in treating stress-induced relapse to opioid misuse and neuropathic chronic non-cancer pain. CBD is a bioactive cannabinoid found in marijuana (*Cannabis sativa*) with low reinforcing properties and anxiolytic-like effects (39–42). Research in a rodent relapse model suggests that repeated CBD administration (5–20 mg/kg) inhibits cue-induced reinstatement of heroin-seeking behavior. Notably, these effects persisted 2 weeks following its administration, a reduction desirable for heroin relapse prevention (42). No adverse side-effects were noted to account for this finding (42). In addition to its anxiolytic effects, CBD may also reduce the reward-facilitating effect of morphine (43). CBD exerts anxiolytic effects partially through serotonin 5-HT-1A receptor activation in the dorsal periaqueductal gray (41). Blockade of 5-HT-1A receptors by a 5-HT-1A receptor antagonist reversed CBD’s inhibition of morphine’s reward-facilitating effects, suggesting that 5-HT-1A receptors may play a role not only in CBD’s anxiolytic properties but also in its opioid reward-inhibiting effects (43). These preclinical findings suggest that CBD, perhaps acting through 5-HT-1A receptor activation to reduce anxiety-related opioid use, may be a promising therapeutic target for opioid use disorder. Pilot data from a human laboratory study in recently heroin-abstinent volunteers with opioid use disorder extended these findings. In these non-treatment seekers, a single administration of oral CBD (400 or 800 mg) blunted cue-induced heroin craving induced by a heroin video cue, compared with placebo. This single administration of CBD maintained a reduction in heroin craving when assessed 24 h later. One hour following a single administration of CBD, CBD also attenuated cue-induced anxiety induced by a heroin cue, compared with placebo (44).

Importantly, a double-blind placebo-controlled cross-over study of CBD, co-administered with an intravenous strong mu-opioid receptor agonist fentanyl, showed that oral CBD (400 or 800 mg) was safe and well tolerated with no significant pharmacokinetic changes with opioid co-administration (45). To extend these preliminary safety and efficacy results, rigorous clinical-trial research is needed with treatment-seeking outpatients having opioid use disorder. For example, it would be helpful to conduct a dose–response study in a double-blind, randomized placebo-controlled trial where recently heroin-abstinent treatment-seeking outpatients would receive a course of one of several doses of CBD or placebo to test CBD’s effectiveness as an opioid relapse-prevention agent. As mentioned, CBD’s inhibition of opiate-mediated reward through activation of serotonin 5-HT-1A receptors may be germane to its effects on blocking opioid reinforcement and reducing anxiety-related susceptibility to opioid use. This hypothesized 5-HT-1A mechanism-of-action merits further systematic and rigorous translational research.

In addition to its anxiolytic effects, preclinical research also suggests that CBD may also have pain-relieving effects in reducing neuropathic pain, partially due to facilitating α3 glycine and serotonin 5-HT-1A receptor activation (46–48), which merits systematic clinical research into this mechanism of action. Furthermore, in placebo-controlled randomized trials, an oromucosal spray formulation [nabiximols, trade name Sativex^®^ approved in the United Kingdom (UK) for treatment of spasticity due to multiple sclerosis] containing about a 1:1 mixture of purified CBD and tetrahydrocannabinol (THC) constituents was found to be efficacious in reducing neuropathic pain (49–51). Phase-III double-blind placebo-controlled randomized trials in United States populations are needed to test the effectiveness of this promising compound for these indications. In addition, an interesting clinically meaningful research question worth studying in a well-designed comparative effectiveness research trial may be the following. Among individuals who have shown signs of misuse while using long-term prescription opioids for neuropathic pain, what are the comparative benefits and harms of continuing conventional opioids (with non-pharmacological co-interventions) versus switching to nabiximols (with or without non-pharmacologic co-interventions)? What is the longer-term durability (>1 year) of these outcomes related to pain, function, and quality of life? Taken together, the above findings suggest multiple potential applications of the cannabinoid CBD in treatment of opioid misuse as well as some forms of chronic non-cancer pain, which merits randomized-controlled trials to test effectiveness, such as the examples discussed above.

## Need for Precision Medicine Approaches to Treating Chronic Non-Cancer Pain, Stress, and Chronic Comorbid Conditions

A key question regarding the putative therapeutic targets and approaches mentioned above would be: for which patient subgroups are the various options most beneficial to maximize therapeutic benefit while minimizing risks for harm, in the broader context of multidisciplinary pain management which may involve psychosocial, shared decision-making co-intervention? Chronic non-cancer pain is a complex neuropsychological syndrome which may have multifaceted biological and psychosocial mediators that may qualitatively differ between distinct patient subgroups (52, 53). Thus, to be useful to clinical practice, additional precision medicine research should be conducted in general medical settings to assess which chronic pain types, related diseases (such as opioid use disorder), and patients are most likely to clinically benefit from such therapeutic options, versus from opioids typically prescribed to treat chronic non-cancer pain. This type of research may also test how to effectively tailor multidisciplinary pain management to individualized pain profiles, patient preferences, risks, and biopsychosocial needs – and tailored to the myriad etiologies and presentations of pain and those of other common co-occurring conditions, such as opioid use disorder. In addition, it would be useful to conduct such comparative effectiveness research in a manner by which patients’ perspectives would be addressed using a shared decision-making approach to evaluate how to improve overall well-being and daily psychosocial functioning from clients’ standpoint and desired outcomes, while simultaneously reducing both psychological distress and physical dimensions of pain (54).

This research area could, thus, also test a range of evidence-based, patient-centered strategies for managing chronic non-cancer pain. Complementary precision medicine research would also be helpful to identify psychiatric, genetic, physiological, sociodemographic, cultural, and other predictors of responsive versus non-responsive patients, incorporating well-accepted and established operational definitions of chronic non-cancer pain (11, 54). Furthermore, rigorous pragmatic research needs to be advanced regarding how to effectively implement a multimodal, team-based collaborative-care approach to chronic pain management in general medical settings, which may involve a multidisciplinary collaboration of physician aids as needed (for example, nurses, psychologists, social workers, pharmacists, etc.) based on comprehensive clinical assessment and biopsychological model of care, working in close coordination to enhance quality and continuity of evidence-based holistic care (54). Such an approach calling for integrated, interdisciplinary team-based pain management is consistent with a need to treat the whole person through a patient-centered care approach, within the context of the entire medical history – including chronic co-occurring conditions – and consistent with personalized needs and preferences, not just the pain condition. This care management approach is also reflective of chronic non-cancer pain not being a single entity or syndrome but being a multidimensional problem and recognizing a need to systematically address in treatment plans the patients’ comorbid conditions, goals of care, recovery and overall well-being, and concomitant medications. Importantly, precision medicine research should include validated clinically meaningful research assessments for identifying patient-reported outcomes on risks and benefits from chronic opioid use, which may be customized to different patient subgroups in general medical settings (11, 54). It would be helpful to assess the degree to which such research assessments accurately predict the risks of opioid misuse, addiction, or overdose. Findings from prospective randomized-controlled trials in the aforementioned priority areas could be supplemented by informative longitudinal data from large pain patient registries linked to electronic health record systems (EHRs), in order to accelerate hypothesis generation and refinement to generate timely knowledge within a rapid-learning healthcare system (54).

In conclusion, considerable neurobiological overlaps exist in neuroadaptations mediating negative psychological components of pain and motivational impact of stress in maintaining or reinstating opioid addictive behaviors. These similarities call for a systematic precision-medicine research agenda, such as the one described above, on how to effectively tailor management of co-occurring chronic non-cancer pain and opioid use disorders in a patient-centered manner using a shared decision-making, multidisciplinary approach. For this approach to address a person’s holistic needs during recovery, care management may take account of patients’ complete medical history, including anxiety, mood, and other psychiatric comorbidities, readiness to change health-risk behaviors and reasons or hesitancy for doing so, and incorporating these factors into treatment plans to support self-management behaviors toward recovery. Pragmatic research may also be conducted to examine how to address patients’ perceived barriers for recovery, as well as individualized values and preferences for treatment options, toward facilitating health promotion behaviors for improving overall functioning and well-being. Such precision medicine type research could bear fruit in establishing which patient populations benefit most from different pain- and substance-use care management regimens. Thereby, this pragmatic clinical research may pave the way to develop evidence-based, actionable clinical decision support tools embedded in EHRs for person-centered approaches to chronic pain management (and common co-occurring conditions), and proactive risk identification and mitigation strategies, in clinical practice.

## Author Contributions

Dr. UG conducted a systematic review of the literature, which he synthesized into the focus of this mini-review, and wrote and reviewed all drafts of this paper.

## Conflict of Interest Statement

The author declares that the research was conducted in the absence of any commercial or financial relationships that could be construed as a potential conflict of interest.
